# Local Bonding Influence on the Band Edge and Band Gap Formation in Quaternary Chalcopyrites

**DOI:** 10.1002/advs.201700080

**Published:** 2017-05-22

**Authors:** Anna Miglio, Christophe P. Heinrich, Wolfgang Tremel, Geoffroy Hautier, Wolfgang G. Zeier

**Affiliations:** ^1^ Institute of Condensed Matter and Nanosciences (IMCN) Université catholique de Louvain Louvain‐la‐Neuve 1348 Belgium; ^2^ Institut für Anorganische und Analytische Chemie Johannes‐Gutenberg‐Universität Duesbergweg 10‐14 Mainz 55099 Germany; ^3^ Physikalisch‐Chemisches Institut Justus‐Liebig‐Universität Giessen Heinrich‐Buff‐Ring 17 Giessen 35392 Germany

**Keywords:** band engineering, chalcopyrites, local bond influence, photovoltaics, thermoelectrics

## Abstract

Quaternary chalcopyrites have shown to exhibit tunable band gaps with changing anion composition. Inspired by these observations, the underlying structural and electronic considerations are investigated using a combination of experimentally obtained structural data, molecular orbital considerations, and density functional theory. Within the solid solution Cu_2_ZnGeS_4−_ *_x_*Se*_x_*, the anion bond alteration parameter changes, showing larger bond lengths for metal–selenium than for metal–sulfur bonds. The changing bonding interaction directly influences the valence and conduction band edges, which result from antibonding Cu–anion and Ge–anion interactions, respectively. The knowledge of the underlying bonding interactions at the band edges can help design properties of these quaternary chalcopyrites for photovoltaic and thermoelectric applications.

## 1 Introduction

Quaternary chalcopyrites Cu_2_M^II^M^IV^Q_4_ (M^II^ = Zn, Fe; M^IV^ = Sn, Ge; Q = S, Se) have attracted interest, nearly quite as much as the ternary analogues, as materials for multiple applications such as photovoltaics, photocatalysts, and thermoelectrics. Especially, the adjustable band gaps in the absorbance region of the solar spectrum lead to good photovoltaic and photocatalytic efficiencies.^1^, ^2^, ^3^, ^4^ In the field of thermoelectrics, the materials have been mostly studied due to their very low thermal conductivities^5^, ^6^, ^7^, ^8^, ^9^, ^10^ and recently discovered band convergence,^11^, ^12^, ^13^ leading to good mid‐temperature range efficiencies.

The interrelation of the structural arrangement and the elemental composition with the band structure and band gaps has led to a good understanding of these materials, especially in the ternary chalcopyrites. While structural disorder shows some influence on the band gap,^14^, ^15^ the cation composition and electronegativity differences mostly determine the band gaps and valence band alignments.^16^, ^17^, ^18^, ^19^, ^20^, ^21^, ^22^, ^23^, ^24^, ^25^ Changing the cation content leads to observable optical bowing,^25^, ^26^, ^27^ i.e., a nonlinear variation of the band gap with composition, whereas changing anions in the chalcopyrite lattice shows little or no optical bowing at all,^10^, ^23^, ^24^, ^28^, ^29^ which makes engineering of the band gap and band edges possible.

Jaffe and Zunger^24^ established a good understanding for the underlying reasons of the observed band gap anomalies in the ternary chalcopyrites, in which three main parameters affect the electronic structures: (1) The cation electronegativity influences the band gap. When cation substitution occurs, the different charge separation changes the energy of the atomic orbitals and therefore the overlap contributions.^24^, ^30^, ^31^ However, the influence of electronegativity is considerably smaller than the influence of p–d‐hybridization.^24^ (2) The degree of p–d‐hybridization between the metal d‐ and anion p‐orbitals determines the valence band energies and band offsets,^24^, ^32^, ^33^, ^34^, ^35^, ^36^ in which a good mixing ensures more metal d‐states being located at the valence band edge.^24^ This mixing is mainly controlled by the energy levels of the atomic orbitals of the constituents, and changing bonding interactions such as bond angles as well as bond distances directly affect this degree of hybridization.^24^ (3) Therefore, a structural effect plays a predominant role in determining the valence band energies. This effect can be directly described by the anion displacement in the structure.^24^, ^25^ On one hand, the anion positions in the ternary and quaternary compounds determine the *c*/2*a*‐ratio of the tetragonal unit cell, with *c*/2*a*‐ratio <1, directly influencing the crystal field splitting of the valence band states in which the splitting decreases linearly with increasing *c*/2*a*‐ratio.^13^, ^24^ On the other hand, the anion position determines the metal–anion bond distance and therefore the orbital overlap, bond strength, and p–d‐orbital mixing.^24^, ^25^, ^30^, ^31^ This so‐called bond alteration parameter, i.e., changing bond length with changing composition, predominantly controls the band gap in ternary chalcopyrites.^24^, ^25^

Inspired by the seminal work of Jaffe and Zunger,^24^ we have explored the band gap behavior in the quaternary solid solution Cu_2_ZnGeS_4−_ *_x_*Se*_x_*. As recently shown,^10^ a linear decrease of the band gap can be observed when replacing S with Se. This is remarkable as Se is slightly more electronegative, which intuitively should result in a larger band gap. Using Jaffe and Zunger's approach for the influence of the bond alteration parameter on the electronic structure,^24^, ^25^ we investigate the dependence of the electronic structure on the structural changes in this solid solution. With changing anion content, the anions are displaced, leading to larger bond lengths for the selenides compared to the sulfide. A combination of density functional theory, molecular orbital considerations, and experimental structural data shows that the increasing bond lengths lead to decreasing bonding interactions and a subsequent reduction in the band gaps in these quaternary chalcopyrites. Understanding the bonding influences on the band edges is a stepping‐stone to designing and engineering chalcopyrites for better thermoelectric and photovoltaic performances.

## 2 Results and Discussion

### 2.1 Structural Considerations

**Figure 1:**
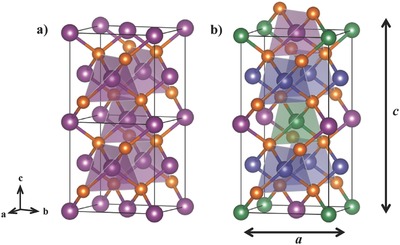
Crystal structures of a) sphalerite ZnQ and b) stannite Cu_2_ZnGeQ_4_ in polyhedral representation showing the anions (Q = S, Se) in orange, Zn in purple, Cu in blue, and Ge in green. The structure of ZnS is shown with two unit cells for a better comparison to the quaternary chalcopyrite, which results from a doubling of the unit cell and subsequent metal ordering. Whereas, the anion is located at (^1^/_4_, ^1^/_4_, ^1^/_4_) in the cubic structure (a), the doubling of the unit cell and the different metals displace the anion position to (*u*, *u*, ^1^/_8_).

In order to follow the discussion of anionic positions as a function of the anion as well as the consequences to the electronic structure, the well‐known general structural evolution of the quaternary chalcopyrites needs to be understood. **Figure** **1** shows ZnQ in the sphalerite structure and the quaternary chalcopyrite Cu_2_ZnGeQ_4_ (Q = S, Se). Both structures derive from the diamond structure and four nearest neighbors coordinate each metal, forming a tetrahedral network structure.^37^ The binary compounds (e.g., ZnS, ZnSe) crystallize in the cubic–sphalerite structure type (space group $F\bar{4}3m$) with anion Wyckoff 4*c* position at (^1^/_4_, ^1^/_4_, ^1^/_4_). Lowering the symmetry by ordered cation substitution with two metals leads to the chalcopyrite structure type with space group $I\bar{4}2d$ and the mineral chalcopyrite CuFeS_2_ as the prototype. This ordered substitution doubles the translational period along the *z*‐direction, leading to a tetragonal crystal system. Due to different bonding interactions between the metals and anions, resulting in different bond lengths and bond angles, a tetragonal distortion takes place resulting in a *c*/2*a*‐ratio <1.^12^, ^13^, ^24^ Higher‐order substitutions, for instance, by tripling or quadrupling the sphalerite lattice, yield compounds such as CuSnSe_3_ and Cu_3_SbSe_4_. Whereas, the doubling of the unit cell from ZnS to CuFeS_2_ changes the anion position in the *z*‐direction from ^1^/_4_ to ^1^/_8_, the *x*‐ and *y*‐position are displaced away from ^1^/_4_ because of the different metals and their corresponding bond lengths.^30^, ^31^ In other words, once two metals with distinct bond lengths are pulling on the anion, the anion positions are shifted in order to minimize the occurring forces.^24^, ^25^

A further decrease in symmetry from the chalcopyrite structure leads to the quaternary stannite structure type (space group $I\bar{4}2m$) or (with further reduced symmetry via metal ordering) the kesterite structure type (space group $I\bar{4}$).^37^, ^38^, ^39^ This decrease in symmetry is achieved by substitution of elements as well as metal ordering. Whereas in the stannite type Cu_2_ZnGeQ_4_, Cu is located on the Wyckoff position 4*d* (0, ^1^/_2_, ^1^/_4_), Zn occupies 2*a* (0, 0, 0), and Ge 2*b* (0, 0, ^1^/_2_). In the kesterite structure, however, Cu is located on the Wyckoff site 2*a* and 2*c* with equal occupancy and Zn occupies the 2*d*‐site. In other words, in the stannite structure Zn and Ge share the *z* = 0 and *z* = ^1^/_2_ metal layers and Cu occupies the *z* = ^1^/_4_ and *z* = ^3^/_4_ layer only, in kesterite all layers are occupied with Cu sharing the layer either with Zn or Ge.

It is impossible to distinguish between the kesterite and stannite structure type by X‐ray diffraction, unless anomalous X‐ray diffraction is used.^40^ Only the Zn^2+^ and Cu^+^ site ordering is different and both elements have the same X‐ray form factor.^9^, ^38^ Therefore, the following discussion on the structural changes in Cu_2_ZnGeS_4−_ *_x_*Se*_x_* is based on the stannite structure. While this assumption may or may not be correct, the structure type does not affect the obtained structural data of the unit cell and anion position, because Zn^2+^ and Cu^+^ are indistinguishable for X‐rays. However, for completion, the density functional calculations were performed for Cu_2_ZnGeS_4−_ *_x_*Se*_x_* in the stannite as well as in the kesterite structure type. In the following sections, the data obtained via density function theory on the stannite structure are used to compare to the experimental structural data. The data calculated for the kesterite structure type can be found in the Supporting Information and confirm the trends observed for the stannite data.

**Figure 2:**
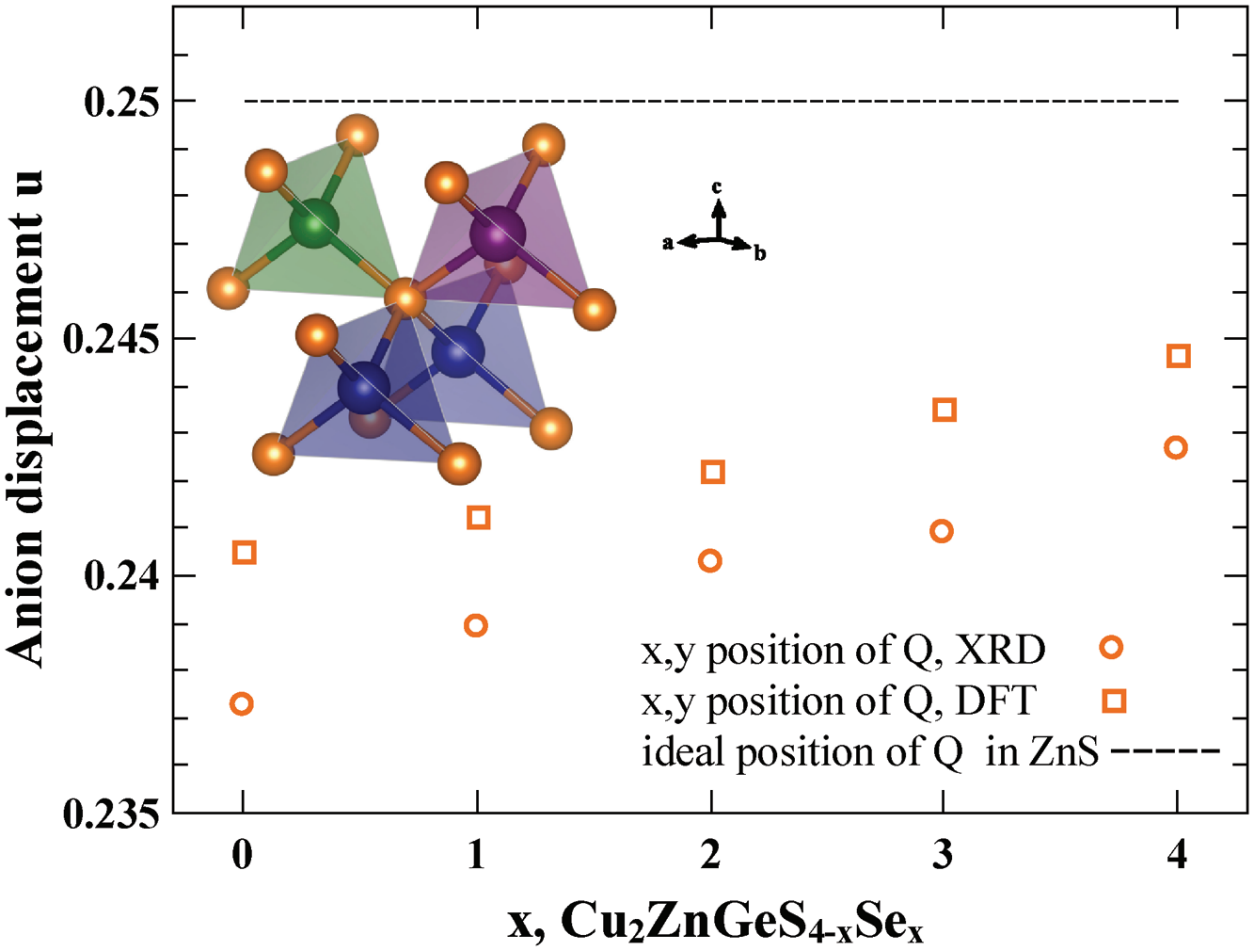
Anion position *u* at Wyckoff position 8*i* (*u*, *u*, ^1^/_8_) in the solid solution Cu_2_ZnGeS_4−_ *_x_*Se*_x_*, obtained from Rietveld refinements against X‐ray diffraction data^10^ (denoted XRD) as well as the ones obtained via density functional theory (denoted DFT). With increasing Se fraction in the structure, the less displaced the anion becomes from the perfect cubic position of *u* = ^1^/_4_. The inset shows a local structural representation of the tetrahedra in the stannite structure. While there is a systematic offset between the calculated and experimentally obtained positions, the general trend is consistent.

**Figure** **2** shows the changing displacement parameter *u* of the anion position (*u*, *u*, ^1^/_8_) with changing anion content, obtained via Rietveld refinement and density functional theory calculations. Both, density functional theory and the experimentally obtained structural data, show similar trends. The observed deviations between the experimental data and the theoretically obtained results correspond to experimental uncertainties of the X‐ray diffraction, as well as the employed functional and known inaccuracy of the absolute values in density functional theory. A higher S content leads to a larger displacement away from *u* = ^1^/_4_, at the anion special position in sphalerite, the increasing amount of Se lowers the displacement. The displacement parameter *u* (*u* < ^1^/_4_) may indeed be the reason why chalcopyrites are able to form solid solutions on the anion site. The displacement lowers the symmetry and the anion position, now a general crystallographic position, can account for any changes in the local bond lengths when larger or smaller anions are introduced.^30^, ^31^

**Figure 3:**
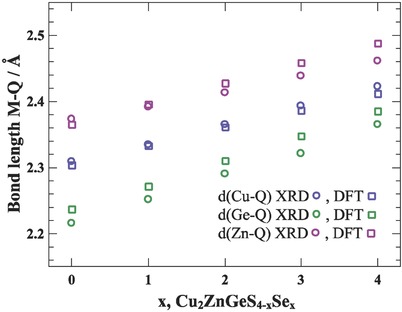
Bond length of the different metal (Cu, Zn, Ge)–anion bond lengths against the fraction of *x* in Cu_2_ZnGeS_4−_ *_x_*Se*_x_*. The increasing Se content leads to increasing *u* and with it an increase in the bond length can be observed, corresponding to Vegard behavior with a larger Se^2−^ radius compared to S^2−^.^41^

**Figure** **3** shows the obtained metal–anion bond lengths of the different compositions in the solid solution Cu_2_ZnGeS_4−_ *_x_*Se*_x_*. With increasing *x*, the displacement *u* increases toward *u* = ^1^/_4_ and the M—Q bond lengths increase, corresponding to the increasing ionic radii from S^2−^ to Se^2−^ and the expected behavior after Vegard.^10^, ^41^ As an increase in the bond distance reduces the orbital overlap and with it the bond strength,^30^, ^31^ which has shown to have influence on the band edges in the ternary chalcopyrites,^24^ the question arises if and how these changing bonding interactions along the Cu_2_ZnGeS_4−_ *_x_*Se*_x_* series affect the electronic properties.

### 2.2 Band Gap Movement

**Figure 4:**
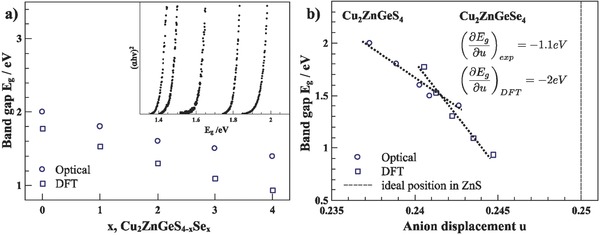
a) Experimentally observed and calculated optical band gaps *E*_g_ in Cu_2_ZnGeS_4−_ *_x_*Se*_x_*. With increasing Se content, the band gap shrinks linearly. The inset shows the experimental optical band gap data as published by Heinrich et al.^10^ b) Dependence of the optical band gaps with the anion displacement, showing the direct effect of a changing displacement on the band gap.

**Figure** **4**a shows the changing band gaps along the series of solid solutions Cu_2_ZnGeS_4−_ *_x_*Se*_x_*, with a decreasing band gap when substituting S with Se. The calculated band gaps are slightly larger using the Heyd–Scuseria–Ernzerhof (HSE) functional, but the general trend of decreasing band gap with increasing selenium content can be observed. Figure 4b shows the experimental rate of changing band gap with displacement $\frac{\partial E_{g}}{\partial u}$ = − 1.1 eV (2.2 eV for the calculated trend) as a descriptor of how much the changing bond length influences the band gap. The rate of changing bond length is slightly larger than that found in the ternary compounds,^25^ showing the direct influence of the bond length on the band gap. As the increasing bond length of the metal–anion bond from sulfur to selenium results in a decrease of the band gap, the p–d‐hybridization is bound to change.^24^

### 2.3 Electronic Structure and Bonding Influences

**Figure 5:**
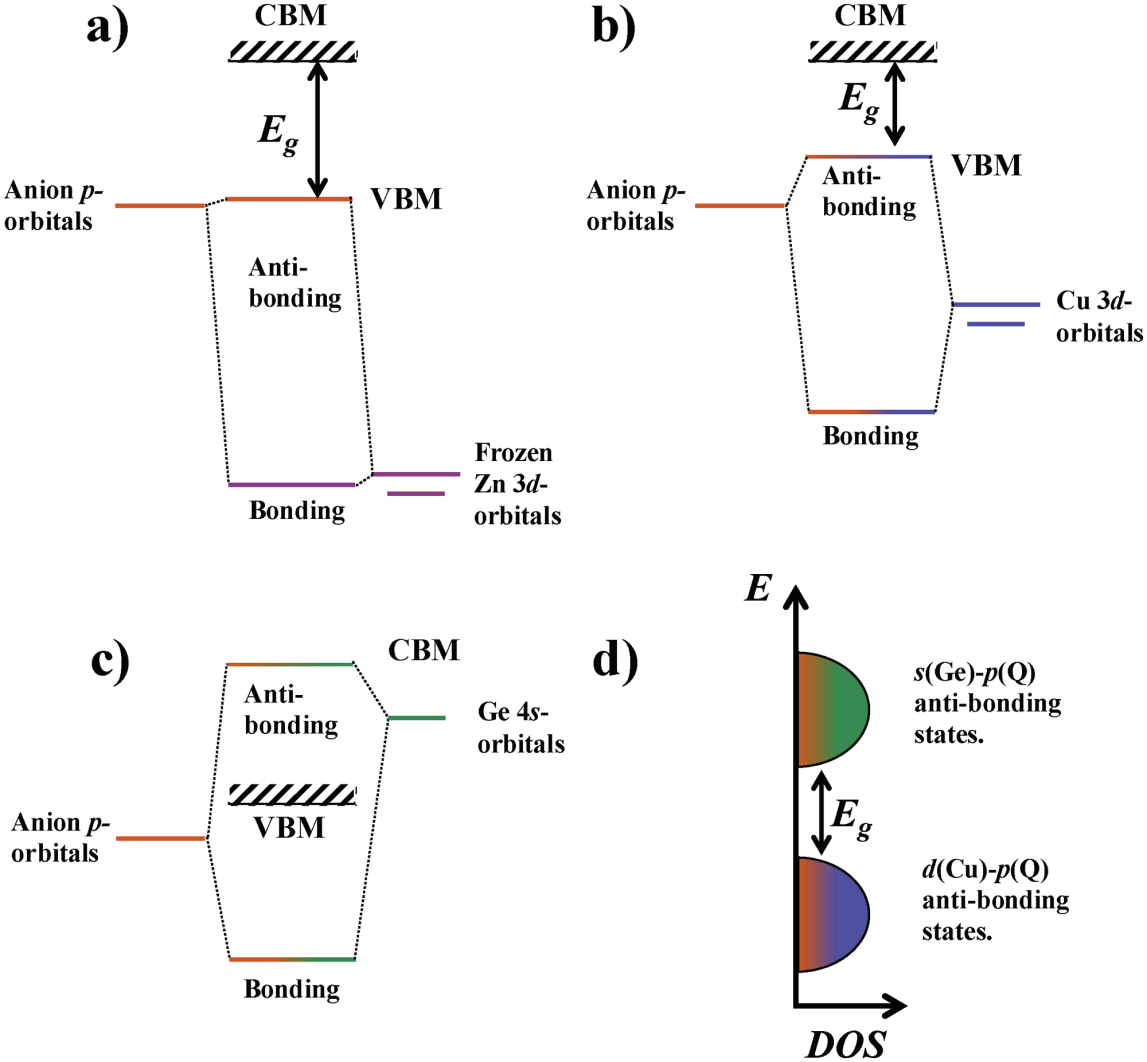
Schematic molecular‐orbital diagram showing the bonding influences of the metal–anion interaction on the band edges. a) The Zn d‐states are low in energy and can be assumed frozen, i.e., no d–p‐bonding interaction can be expected. b) The Cu 3d atomic orbitals are closer in energy to the anion p‐orbitals resulting in orbital overlap and a significant bonding interaction. The p–d‐hybridized antibonding state forms the valence band edge. c) The empty Ge 4s atomic orbitals form bonding interactions with the anion p‐states, and the antibonding states can be expected in the conduction band edge. Therefore, changes to the Cu—Q and Ge—Q interactions are bound to change the energy separation between the bonding and antibonding states, directly influencing the band gap. d) Schematic p density of states (DOS) with Cu—Q bonding states as the valence band edge and Ge—Q antibonding states as the conduction band edge.

**Figure 6:**
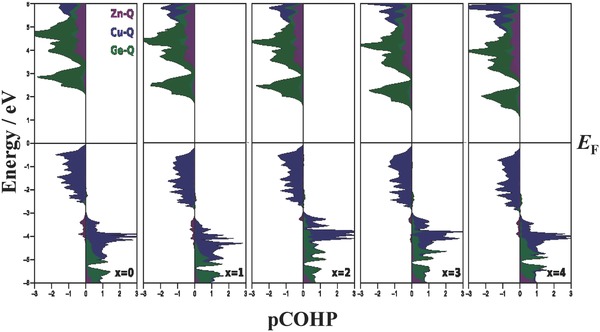
Crystal Orbital Hamilton populations (COHP) of stannite Cu_2_ZnGeS_4−_ *_x_*Se*_x_* as a function of the anion content *x*. While the Zn—Q states are low in energy in the valence band, the valence band edge is dominated by antibonding Cu 3d–Q p interactions. The conduction band edge on the other hand is primarily composed of Ge 4s–Q p interactions that are of antibonding nature. With increasing amount of Se, both valence and conduction bands move lower in energy, however, the change in energy is higher for the conduction band.

In order to understand the direct influence of the bond length and p–d‐hybridization on the band structure and optical band gaps, **Figure** **5** shows a schematic molecular orbital diagram of the different bonding interactions in Cu_2_ZnGeQ_4_. As discussed by Jaffe and Zunger for ternary compounds,^24^ the metal–anion bonding interactions can be used to describe the valence band edge. On one hand, the Zn 3d‐orbitals should be low in energy and no significant orbital interaction or p–d‐hybridization with the S and Se p‐states can be expected. The Cu 3d‐states, on the other hand, are higher in energy and therefore contribute to the band edge energies.^16^, ^34^, ^35^ This Cu–anion bonding interaction leads to bonding states in the valence bands and antibonding states with Cu–anion character at the valence band edge. As recently proposed,^42^ **Figure** **6** shows calculated Crystal Orbital Hamiltonian Populations (COHP)^43^ for Cu_2_ZnGeQ_4_ in the stannite structure along the series of solid solutions, confirming the Cu—Q antibonding character at the valence band edge, low lying Zn 3d‐states and low lying Cu—Q bonding states. As discussed by Walsh and co‐workers,^3^ the conduction band minimum in these quaternary chalcopyrites can be expected to be of antibonding Ge—Q character (Figure 5c). The empty Ge 4s‐orbitals interact with the anion p‐states to form bonding states in the valence band and antibonding states at the conduction band edge, which can also be observed in the COHP of the stannite structure (Figure 6). This calculated COHP for the kesterite structure type shows similar energetic arrangements of the bonding and antibonding states and can be found in the Supporting Information. Therefore, a qualitatively correct discussion of the bonding interactions and the electronic structure is expected, even if the structure exhibits different cation ordering.

The understanding of Ge—Q antibonding states at the conduction band edge and Cu—Q antibonding states at the valence band edge provides some insight into how the band energies change with changing bonding interaction, because the band energies and density of states can be directly inferred from any molecular orbital interactions.^30^, ^31^, ^44^, ^45^ In general, a stronger bonding interaction will increase the energy separation between the bonding and antibonding states. Therefore, an increasing Cu—Q bonding interaction will shift the antibonding state (Figure 5b), i.e., the valence band maximum to higher energies and vice versa, a decreasing Cu—Q bonding interaction will lead to a lowering of the valence band edge. In other words, a decreasing Cu—Q bonding interaction should lead to a lowering of the valence band edge and an increase in the band gap. As Jaffe and Zunger have shown,^24^, ^25^ this is indeed the reason for the changing band gaps in materials such as CuGaSe_2_ or CuAlS_2_, in which the low lying states have no major influence. In the case of the quaternary compounds, however, the Ge—Q antibonding interaction at the conduction band minimum needs to be considered as well (Figure 5c). With decreasing bonding interaction, the conduction band minimum is expected to shift to lower energies, in turn decreasing the band gap of the material.

**Figure 7:**
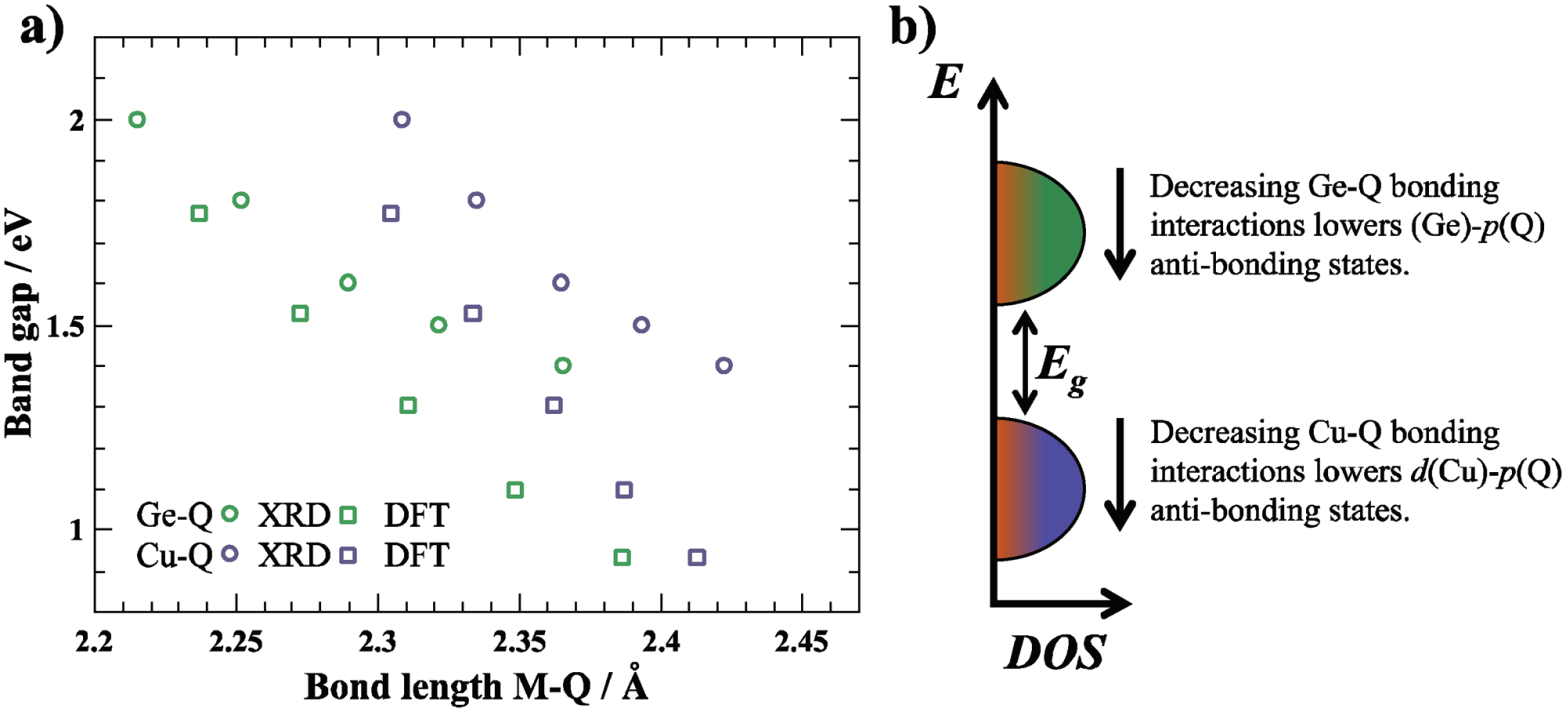
a) Experimentally observed and calculated band gap *E*_g_ against the bond length of the Cu—Q and Ge—Q bonds, showing the decrease of the band gap with increasing bond length. b) Schematic density of states (DOS) of the band edge contributions and the band gap. The increasing bond lengths from S to Se lead to a decreasing bonding interaction between Cu—Q and Ge—Q and a concurrent decrease of the valence band maximum and conduction band minimum. The Ge—Q bond decreases faster along the series of solid solutions, which ultimately leads to a reduction of the band gap when substituting S with Se.

In other words, the substitution of S with Se leads to an increase in the Cu—Q and Ge—Q bond lengths (see Figure 3), which reduces the bonding interactions along the series of solid solutions Cu_2_ZnGeS_4−_ *_x_*Se*_x_*. Therefore, moving from S to Se will lead to a decrease of the conduction band minimum as the antibonding Ge s‐state–Q p‐state shift to lower energies. In addition to shifting the conduction band minimum, the valence band maximum will concurrently shift to lower energies due to the decreasing bonding interaction of Cu d with Q p. These considerations show, that an increasing bond length and decreasing bonding interactions will shift both valence and conduction band edges to lower energies, which raises the question why the band gap changes at all. **Figure** **7**a shows the dependence of the band gap on the bond lengths of Cu—Q and Ge—Q and a schematic for the density of states is shown in Figure 7b. While both band edges decrease in energy with increasing bond lengths, the Ge—Q bond expands much faster and more than the Cu—Q bond, in turn leading to a faster decrease of the conduction band minimum compared to the valence band maximum.

In addition to the changing bond lengths, the substitution of S with Se has shown to distort the tetrahedral connectivity and forcing the M—Q—M bond lengths to a more perfect tetrahedral angle of 109.5°.^10^, ^30^ This changing bond angle as well as the increasing overlap of the larger Se^2−^ anion (when considering p–p and p–d overlap) should lead to a broader valence band dispersion, which should affect the effective mass and carrier mobility. This effect has indeed been observed for Cu_2_ZnGeS_4−_ *_x_*Se*_x_* experimentally,^7^, ^10^ and to a similar extent in Cu_2−_ *_x_*Se.^46^, ^47^

Knowing the contributions and bonding interactions at the valence and conduction band edge helps to design quaternary chalcopyrites for better performing photovoltaics or thermoelectrics. Doping studies can be designed in order to not disrupt charge transport in the respective band. For instance, in order to not detrimentally affect the hole mobility, doping studies should mainly focus on substitution of Ge as it will only affect the conduction band.^48^

## 3 Conclusion

In summary, we have extended the description of influences of crystal structure and bonding interactions of chalcopyrites from the ternary to the quaternary compounds. While the d–p‐hybridization between the metal and anions determine the valence band edges, the Ge s–anion p interactions influence the conduction band edge. Using a combination of experimentally obtained structural data with molecular orbital considerations and density function theory, it is possible to understand occurring electronic changes in the quaternary chalcopyrites.

The increasing anion radius changes the bond lengths and with it the overlap contributions, which lead to a decrease of the valence band maximum and conduction band minimum. Due to the faster changing bond lengths that affect the conduction band, the conduction band minimum is moved to lower energies much faster than the valence band, leading to the observed decrease in the band gap.

This work shows how bonding interactions, such as bond length, anion size, and bond angles affect the band edge and with it the inherent electronic transport. Knowing the contributions and bonding interactions at the valence and conduction band edge will guide future research when designing new semiconductors and compositions and helps to design quaternary chalcopyrites for better photovoltaics or thermoelectrics.

## 4 Experimental Section

*Synthesis and Structural Data*: Synthesis as well as X‐ray diffraction data collection, and corresponding Rietveld refinements of the polycrystalline stannite‐type Cu_2_ZnGeS_4−_ *_x_*Se*_x_* had been reported elsewhere.^10^ For obtaining anion displacement and bond lengths, the refined atomic positions and lattice parameters had been used as input in the structural program VESTA 3.^49^

*Density Functional Theory*: Density functional theory (DFT) calculations were performed with the projector augmented wave^50^ approach as implemented in the Vienna Ab Initio Simulation Package (VASP).^51^, ^52^ A planewave cutoff of 520 eV and a mesh of 6 × 6 × 4 k‐points were used. Standard DFT does not describe correctly the Cu d–anion p hybridization and leads to the incorrect description of both electronic and structural properties. Indeed, DFT severely underestimates the band gap and the anion displacement in chalcopyrites. The use of the HSE^53^ hybrid functional can overcome these limitations.^54^ On the HSE results, orbital overlap was analyzed within the COHP framework as implemented in the lobster software.^43^, ^55^, ^56^

## Conflict of Interest

The authors declare no conflict of interest.

## Supporting information

Supplementary
