# Supplementary material for: Local Bonding Influence on the Band Edge and Band Gap Formation in Quaternary Chalcopyrites
Source: Adv Sci (Weinh). 2017 May 22;4(9):1700080. doi: 10.1002/advs.201700080 (PMC5604395; doi:10.1002/advs.201700080)
Supplement: Supplementary file 1 — Supplementary [file ADVS-4-na-s001.pdf]

Copyright WILEY-VCH Verlag GmbH & Co. KGaA, 69469 Weinheim, Germany, 2017.

Supporting Information

for *Adv. Sci.,* DOI: 10.1002/advs.201700080

Local Bonding Influence on the Band Edge and Band Gap Formation in Quaternary Chalcopyrites

Anna Miglio, Christophe P. Heinrich, Wolfgang Tremel, Geoffroy Hautier, and Wolfgang G. Zeier**

Copyright WILEY-VCH Verlag GmbH & Co. KGaA, 69469 Weinheim, Germany, 2016.

Supporting Information –

Local bonding influence on the band edge and band gap formation in quaternary chalcopyrites

Anna Miglio, Christophe P. Heinrich, Wolfgang Tremel, Geoffroy Hautier,* and Wolfgang G. Zeier*

[a]*Institute of Condensed Matter and Nanosciences (IMCN)*

Université catholique de Louvain, 1348 Louvain-la-Neuve, Belgium

[b]*Institut für Anorganische und Analytische Chemie, Johannes-Gutenberg-Universität, Duesbergweg 10-14, 55099 Mainz, Germany*

[c]*Physikalisch-Chemisches Institut, Justus-Liebig-Universität Giessen, Heinrich-Buff-Ring 17, 35392 Giessen, Germany*

Table S1: Results from density function calculations (HSE functional) for kesterite $Cu_2ZnGeS_{4-x}Se_x$, giving the different lattice parameters *a*, bond lengths M-Q, total energy E_{tot}, band gap E_g and anion displacement *u*.

x	a / Å	Cu1-Q	Cu2-Q	Ge-Q	Zn-Q	E_{tot} /eV	E_g / eV	u
0	5.339	2.307	2.334	2.233	2.339	-47.80	2.08	0.2398
1	5.400	2.335	2.362	2.271	2.371	-45.50	1.84	0.2408
2	5.483	2.363	2.392	2.308	2.400	-43.24	1.62	0.2417
3	5.546	2.391	2.420	2.344	2.429	-40.99	1.42	0.2427
4	5.628	2.414	2.449	2.385	2.457	-38.77	1.21	0.2442

Table S2: Results from density function calculations (HSE functional) for stannite $Cu_2ZnGeS_{4-x}Se_x$, giving the different lattice parameters *a*, bond lengths M-Q, total energy E_{tot}, band gap E_g and anion displacement *u*.

x	a / Å	Cu1-Q	Cu2-Q	Ge-Q	Zn-Q	E_{tot} /eV	E_g / eV	u
0	5.303	2.304	2.304	2.236	2.365	-47.75	1.77	0.2405
1	5.371	2.333	2.333	2.272	2.396	-45.45	1.53	0.2412
2	5.456	2.361	2.361	2.312	2.429	-43.20	1.33	0.2423

2	5.446	2.361	2.361	2.308	2.427	-43.16	1.27	0.2420
3	5.523	2.386	2.386	2.348	2.458	-40.94	1.09	0.2435
4	5.587	2.412	2.412	2.385	2.487	-38.72	0.93	0.2446

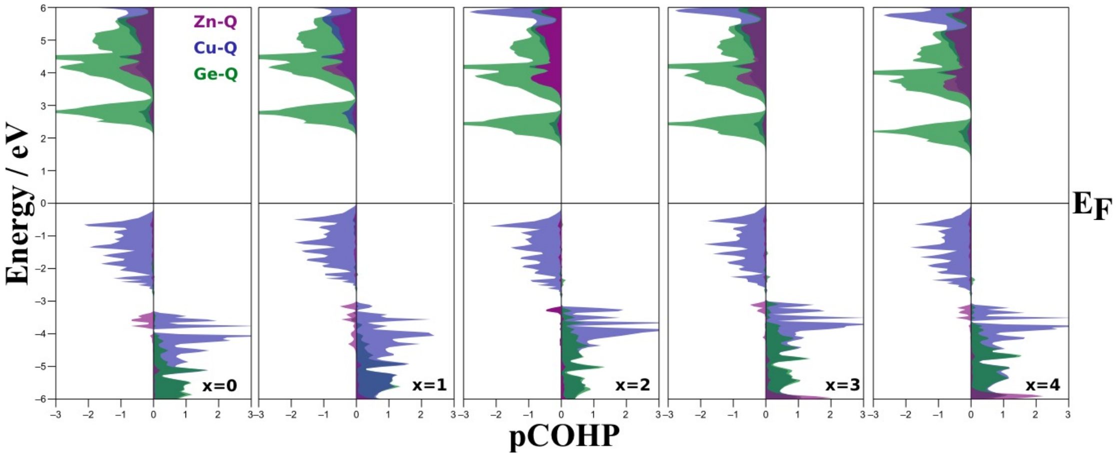

Figure S1: Crystal Orbital Hamilton populations (COHP) obtained from DFT-HSE calculations of kesterite $Cu_2ZnGeS_{4-x}Se_x$ as a function of the anion content x.

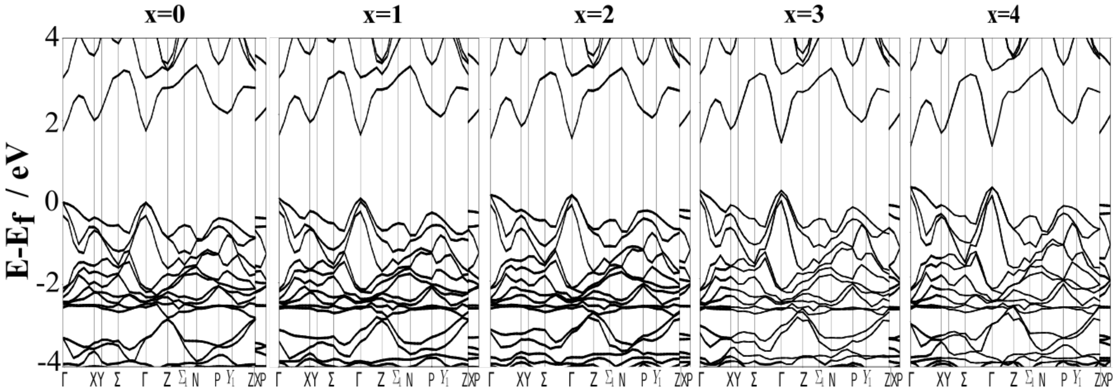

Figure S2: Results for density function calculations (HSE functional) of stannite $Cu_2ZnGeS_{4-x}Se_x$ as a function of the anion content x.

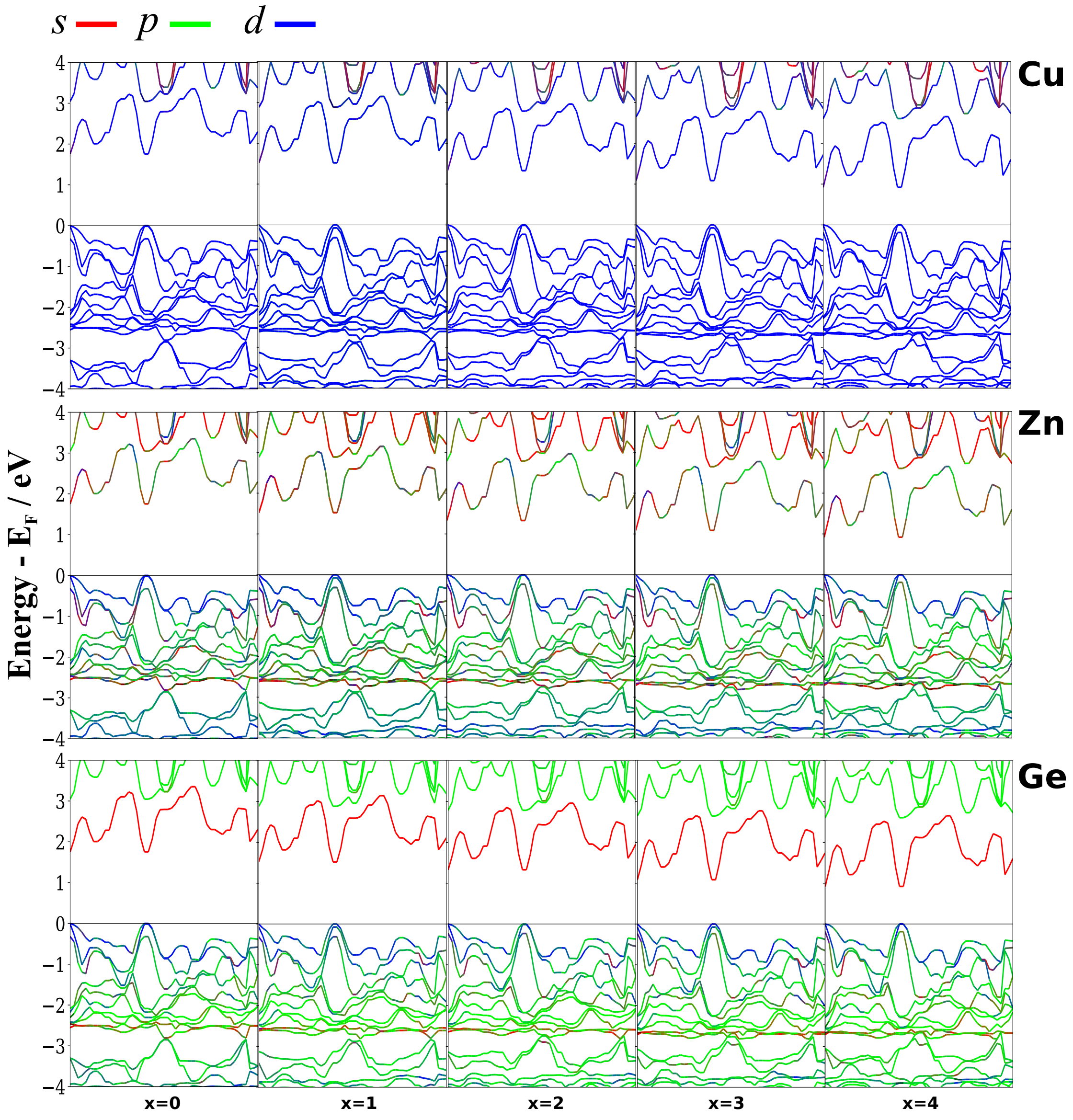

Figure S3: Atom projected results for density function calculations (HSE functional) of stannite $Cu_2ZnGeS_{4-x}Se_x$ as a function of the anion content x.

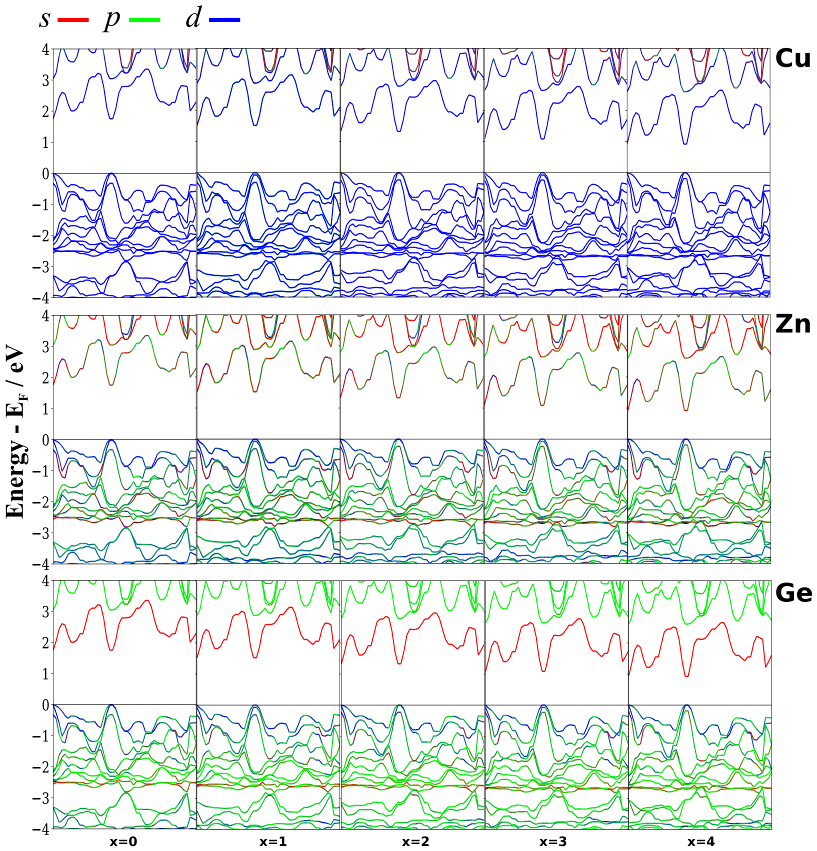

Figure S4: Orbital-projected band structures from density function calculations (HSE functional) of stannite $Cu_2ZnGeS_{4-x}Se_x$ for each cation component and as a function of the anion content x.
